# Renal impairment may indicate postoperative low vision in young patients with proliferative diabetic retinopathy undergoing vitrectomy

**DOI:** 10.3389/fendo.2023.1321226

**Published:** 2024-01-11

**Authors:** Xiaorong Zheng, Lin Feng, Chen Xing, Junlan Wang, Wei Zhao, Fengmei Zhang

**Affiliations:** ^1^ Clinical Laboratory, Hebei Provincial Key Laboratory of Ophthalmology, Hebei Provincial Clinical Research Center for Eye Diseases, Hebei Eye Hospital, Xingtai, Hebei, China; ^2^ Diabetic Eye Disease Ward, Hebei Provincial Key Laboratory of Ophthalmology, Hebei Provincial Clinical Research Center for Eye Diseases, Hebei Eye Hospital, Xingtai, Hebei, China; ^3^ Fundus Imaging and Laser Therapy Department, Hebei Provincial Key Laboratory of Ophthalmology, Hebei Provincial Clinical Research Center for Eye Diseases, Hebei Eye Hospital, Xingtai, Hebei, China; ^4^ Internal Medicine Department, Hebei Provincial Key Laboratory of Ophthalmology, Hebei Provincial Clinical Research Center for Eye Diseases, Hebei Eye Hospital, Xingtai, Hebei, China; ^5^ Medical Records Room, Hebei Provincial Key Laboratory of Ophthalmology, Hebei Provincial Clinical Research Center for Eye Diseases, Hebei Eye Hospital, Xingtai, Hebei, China

**Keywords:** proliferative diabetic retinopathy, renal impairment, vitrectomy, low vision, cystatin C, estimate glomerular filtration rate, urine protein

## Abstract

**Objective:**

To innovatively evaluate the impact of renal impairment in young work age patients with proliferative diabetic retinopathy (PDR) on their visuality after vitrectomy.

**Methods:**

To find out whether it is possible to better predict the improvement of visual acuity after vitrectomy in working-age people without adding additional preoperative testing. In view of the fact that diabetic retinopathy and diabetic nephropathy are common diabetic complications of microvascular damage, it is considered whether preoperative renal function can be used as this evaluation index. This paper studies the design under this theme. This retrospective study included 306 patients (306 eyes) diagnosed with PDR and undergoing vitrectomy in our hospital from January 2016 to June 2023. Relevant baseline data were collected, including age, history of kidney disease and clinical laboratory test results. According to the International Standard Logarithmic Visual Acuity Checklist, the best corrected visual acuity was tested on the first day of admission and one month after surgery, and the difference between the two was subtracted. A difference >0 was defined as “vision improved”. Patients were classified as vision-improved group (n=245) and non-improved group (n=61). The differences in baseline serum urea nitrogen, creatinine, uric acid, Cystatin C, estimated glomerular filtration rate (eGFR) and urine protein distribution between the two groups were statistically analyzed, binary regression analysis was performed for meaningful parameters, and random forest model ranked the characteristics in importance.

**Results:**

1.A higher level of serum cystatin C [1.02(0.80,1.48) mg/L vs 0.86(0.67,1.12) mg/L, P<0.001] and a lower eGFR [82.3(50.33, 115.11) ml/(min/1.73m²) vs 107.69(73.9, 126.01) ml/(min/1.73m²), P=0.002] appeared in the non-(vision-)improved group compared with the vision-improved group. 2. The occurrence of preoperation proteinuria history of nephropathy take a larger proportion in non-improved group. 3. Univariate regression analysis showed history of nephrology (OR=1.887, P=0.028), preoperative serum urea nitrogen (OR=0.939, P=0.043), cystatin C (Cys-C) concentration (OR=0.75, P=0.024), eGFR (OR=1.011, P=0.003) and proteinuria (OR=3.128, P<0.001) were influencing factors to postoperative visual acuity loss in young working age PDR patients. Excluding other confounding factors, preoperative proteinuria is an independent risk factor for postoperative vision improvement in working-age PDR populations (OR=2.722, P=0.009). 4. The accuracy of the prediction random forst model is 0.81. The model appears to be superior in terms of positive prediction.

**Conclusion:**

In young work aged PDR patients undergoing vitrectomy, preoperative urine protein can be an independent indicator of postoperative visual loss. Aggressive correction of kidney injury before surgery may help improve postoperative vision in patients with PDR.

## 1 Introduction

Diabetic patients have abnormal blood glucose elevation in the non-intervention state due to the impaired uptake and utilization of glucose, and the body cells are in a state of high glucose for a long time, which eventually leads to damage to multiple tissues and organs. It can manifest as increased permeability of small vessels, vascular occlusion leading to compensatory neovascularization, rupture and bleeding of fragile neovascularization, and exacerbation of the disease process. Diabetic nephropathy(DN) and diabetic retinopathy (DR) are common complications of diabetes. China has currently the largest number of diabetic patients in the world (1), with one DR for every there DM patients (2). DR is the first blinding eye disease in working-age people, which seriously affects the quality of work and life and has become a major public health problem. DR can be clinically divided into non-proliferative DR (NPDR) and proliferative DR (PDR), the latter requiring surgery in severe cases. Whether postoperative vision can be effectively improved is worthy of more attention. Studies have found that the progression of DR is associated with renal pathological changes and the degree of progression of end-stage renal disease (3). The sensitivity and specificity of DR predicting DN in patients with T2DM is 0.65 and 0.75, separately (4). In view of the link between DR and DN, is it possible to predict postoperative visual acuity changes in young patients from the preoperative assessment of renal function status? This article briefly showed the discussion.

## 2 Methods

### 2.1 Selection of research subjects

Inclusion criteria: (1) patients with diabetes (type 1, type 2); (2) worhing age people (aged 18-55 years); (3) The diagnosis and staging of PDR conform to the “Guidelines for Clinical Diagnosis and Treatment of Diabetic Retinopathy in China (2022)” (5), and the evidence of fundus imaging examination is clear.

Exclusion criteria: (1) Patients with active infection (bacterial or viral infections), autoimmune diseases (eg, lupus, multiple sclerosis, rheumatoid arthritis and psoriasis) and malignant tumors, pregnant and lactating women; (2) Patients with glaucoma, eye trauma, hypertensive retinopathy and other eye diseases; (3) Special types of diabetes caused by immune-mediated type 1 diabetes, pancreatic diabetes, infection, other endocrine diseases, glucocorticoids, anti-tumor drugs, etc;(4) Have received intraocular surgery (excluding photocoagulation therapy) before the visit;(5) Non-diabetic-related kidney disease, such as congenital renal absence or renal hypoplasia, acute/chronic glomerulonephritis, renal cysts, acute kidney injury, urinary tract infection; (6) High-protein, high-purine diet within half a month before the visit.

According to the above criteria, 306 young PDR patients (aged 20-55 years, 306 eyes) who were hospitalized in our hospital and treated with vitrectomy from January 2016 to June 2023 were continuously enrolled.

### 2.2 Research methods

Collected relevant baseline data of the patient’s hospitalization, including age, gender, previous renal history, preoperative clinical serological tests (renal function) and so on. The visual acuity before and after treatment were recorded, and the difference between the two was made. Preoperative visual acuity examination: The best visual acuity that can be achieved in the affected eye after the patient has adequately corrected the patient’s refractive errors (myopia, farsightedness, and astigmatism) before surgery. The International Standard Visual Acuity Chart was used to perform distance visual acuity examination, and the results were recorded using *the Miu method* (5-point expression) (5). Postoperative visual acuity examination: about 1 month after vitrectomy surgery, the gas or liquid to be filled is fully absorbed, and the patient’s best corrected visual acuity is measured and recorded. Study postoperative visual acuity and preoperative visual acuity. Patients with the difference >0 were included in the study group, defined as “vision-improved group”; the difference ≤0 were included in the control group named “non-improved group”. Statistical methods were used to compare whether there was any difference in the distribution of renal function evaluation indexes between the two groups.

### 2.3 Statistical analysis

Firstly, we used SPSS25.0 software for statistical analysis. After all continuous variables were tested for normality, data of normal distribution was described as mean ± standard deviation (X ± SD), and the mean difference between two groups was compared by the t-test of two independent samples. Data of nonnormal distributors was represented by *M (P25, P75)*, and the nonparametric test compared the difference between two groups. The chi-square test compared differences in discontinuous variables between groups. Binary Logistic Regression analyzed the influencing factors of postoperative visual acuity decline in young PDR patients. P<0.05 indicates that the difference is statistically significant. In this study, multiple variables such as age, medical history, and renal function tests were included, and the relationship between these variables was complex, and binary regression analysis alone could not meet the research needs. We could try to use machine learning approaches. Nowadays the machine learning and deep learning models have been used in many fields, including agriculture (6), environment (7–9) and medical field (10). We utilized Random Forest machine learning techniques for the multivariate classification analysis.

## 3 Results

### 3.1 Baseline data

Three hundred and six eyes from306 young PDR patients were included in the study. As shown in **Table 1**, there was no difference between the two groups on gender (p=0.779), history of hypertension (p = 0.288), surgical eyes (p=0.587), mean age (p=0.495), diabetes duration (p=0.649), serum glucose level (p=0.975) and Glycosylated hemoglobin (p=0.573). The two groups showed significant differences on history of hypertension (X^2 ^= 4.896, p = 0.027).

**Table 1:** Baseline characteristics according to vision difference (non-improved group ≤ 0, vision-improved group>0).

index	Total observed (n=306)	vision-improved group (n=245)	non-improved group (n=61)	Z value	P value
Gender〔male, n (%)〕	201 (65.7)	160 (65.3)	41 (67.2)	0.079*	0.779
history of nephrology, n (%)	137 (44.8)	102 (41.6)	35 (57.4)	4.896*	0.027
history of hypertension, n (%)	162 (52.9)	126 (51.4)	36 (59.0)	1.129*	0.288
Surgical eye [left, n (%)]	155 (50.7)	119 (48.6)	32 (52.5)	0.295*	0.587
age, years	38 (33, 46)	38 (34, 44)	40 (31,50)	-0.682	0.495
DM duration, year	6.5 (1.5, 12.0)	6.0 (2.0, 12.0)	8.0 (1.5, 13.0)	-0.456	0.649
Glu, mmol/L	6.69 (5.37, 8.62)	6.70 (5.38, 8.62)	6.60 (5.24, 8.65)	-0.031	0.975
HbAlc, %	7.5 (6.6, 8.9)	7.5 (6.6, 9.0)	7.5 (6.6, 8.5)	-0.564	0.573

*stands for X^2^value, Glu, glucose; DM, diabetes mellitus.

### 3.2 Renal function

#### 3.2.1 Serology test

All 306 patients underwent vitrectomy and were tested to evaluate their renal function on the early morning fasting on second day in hospital. According to **Table 2**, the serum Cys-C and urea nitrogen level in non-improved group was separately significantly higher than that of the vision-improved group [1.02 (0.80, 1.48) mg/L vs 0.86 (0.67, 0.1.12) mg/L, P<0.001; 7.71(5.41, 9.69)mmol/L vs 6.08(4.52, 7.95)mmol/L, p=0.005]. The eGFR of the non-improved group [82.30(50.33, 115.11) ml/(min/1.73m^2^)] was significantly lower than that of the vision-improved group [103.69(67.67, 124.87) ml/(min/1.73m^2^)], P=0.002. There was no significant difference in creatinine and uric acid levels between the two groups (P >0.05).

**Table 2:** Serology test for renal function according to vision difference (non-improved group ≤ 0, vision-improved group>0).

index	Total observed (n=306)	vision-improved group (n=245)	non-improved group (n=61)	Z value	P value
urea nitrogen, mmol/L	6.30 (4.61, 8.61)	6.08 (4.52, 7.95)	7.71 (5.41, 9.69)	-2.838	0.005
creatinine, μmol/L	75 (59, 113)	74 (60, 105)	96 (57, 132)	-1.632	0.103
uric acid, μmol/L	357 (296, 412)	357 (295, 414)	362 (309, 401)	-0.182	0.856
Cys-C, mg/L	0.88 (0.68, 1.22)	0.86 (0.67, 1.12)	1.02 (0.80, 1.48)	-3.672	<0.001
eGFR, ml/(min/1.73m^2^)	103.69 (67.67, 124.87)	107.69 (73.89, 126.01)	82.30 (50.33, 115.11)	-3.031	0.002

#### 3.2.2 Urine test

According to **Table 3**, in young PDR patients undergoing vitrectomy, the rate of preoperative proteinuria in vision-improved group was significantly lower compared to the non-improved group (47.3% vs 73.8%, P<0.001). There was no significant difference in preoperative haematuria, glycosuria and pyuria between the two groups (P >0.05).

**Table 3:** Urine test for renal function according to vision difference (non-improved group ≤ 0, vision-improved group>0).

index	Total observed (n=306)	vision-improved group (n=245)	non-improved group (n=61)	x^2^value	P value
Urine glucose [positive, n (%)]	72 (23.5)	56 (22.9)	16 (26.2)	0.309	0.578
Urine protein [positive, n (%)]	161 (52.6)	116 (47.3)	45 (73.8)	13.677	<0.001
Urine RBC [positive, n (%)]	91 (29.7)	69 (28.2)	22 (36.1)	1.46	0.227
Urine WBC [positive, n (%)]	25 (8.2)	18 (7.3)	7 (11.5)	0.628	0.428

RBC, red blood cells; WBC, white blood cells.

### 3.3 Logistic regression analysis

As shown in **Table 4**, univariate regression analysis was performed for all variables, and We selected variables with P<0.1 for inclusion in the next round of multivariate regression analysi, including history of nephrology (OR=1.887, P=0.028), preoperative serum urea nitrogen (OR=0.939, P=0.043), cystatin C concentration (OR=0.75, P=0.024), eGFR (OR=1.011, P=0.003) and proteinuria (OR=3.128, P<0.001). The results showed that only one variable was left for last. Excluding other confounding factors, preoperative proteinuria is an independent risk factor for postoperative vision improvement in working-age PDR populations (OR=2.722, P=0.009).

**Table 4:** Logistic regression analysis for factors influencing postoperative vision decrease.

Index	Univariate analysis		Multivariate analysis	
	P value	OR (95%CI)	P value	OR (95%CI)
Gender〔male, n (%)〕	0.779	1.089 (0.6, 1.976)		
history of nephrology, n (%)	0.028	1.887 (1.07, 3.329)	0.752	0.881 (0.4, 1.939)
history of hypertension, n (%)	0.289	1.36 (0.77, 2.401)		
Surgical eye [left, n (%)]	0.587	0.856 (0.488, 1.501)		
age, years	0.546	0.99 (0.957, 1.023)		
DM duration, year	0.585	0.988 (0.945, 1.033)		
Glu, mmol/L	0.95	0.997 (0.913, 1.089)		
HbAlc, %	0.514	1.058 (0.893, 1.254)		
Serum urea nitrogen (mmol/L)	0.043	0.939 (0.884, 0.998)	0.567	1.030 (0.93, 1.142)
Serum creatinine (μmol/L)	0.206	0.999 (0.996, 1.001)		
Serum uric acid (μmol/L)	0.984	0.999 (0.997,1.003)		
Serum Cys-C (mg/L)	0.024	0.75 (0.584,0.962)	0.379	0.855 (0.603, 1.212)
eGFR [ml/ (min/1.73m2)]	0.003	1.011 (1.004, 1.019)	0.514	1.005 (0.989, 1.021)
Urine glucose, positive	0.579	1.2 (0.63, 2.284)		
Urine protein, positive	<0.001	3.128 (1.677,5.833)	0.009	2.722 (1.289,5.746)
Urine RBC, positive	0.228	1.439 (0.796,2.601)		
Urine WBC, positive	0.296	1.635 (0.65, 4.111)		

### 3.4 Random forest model

**Figure 1:**
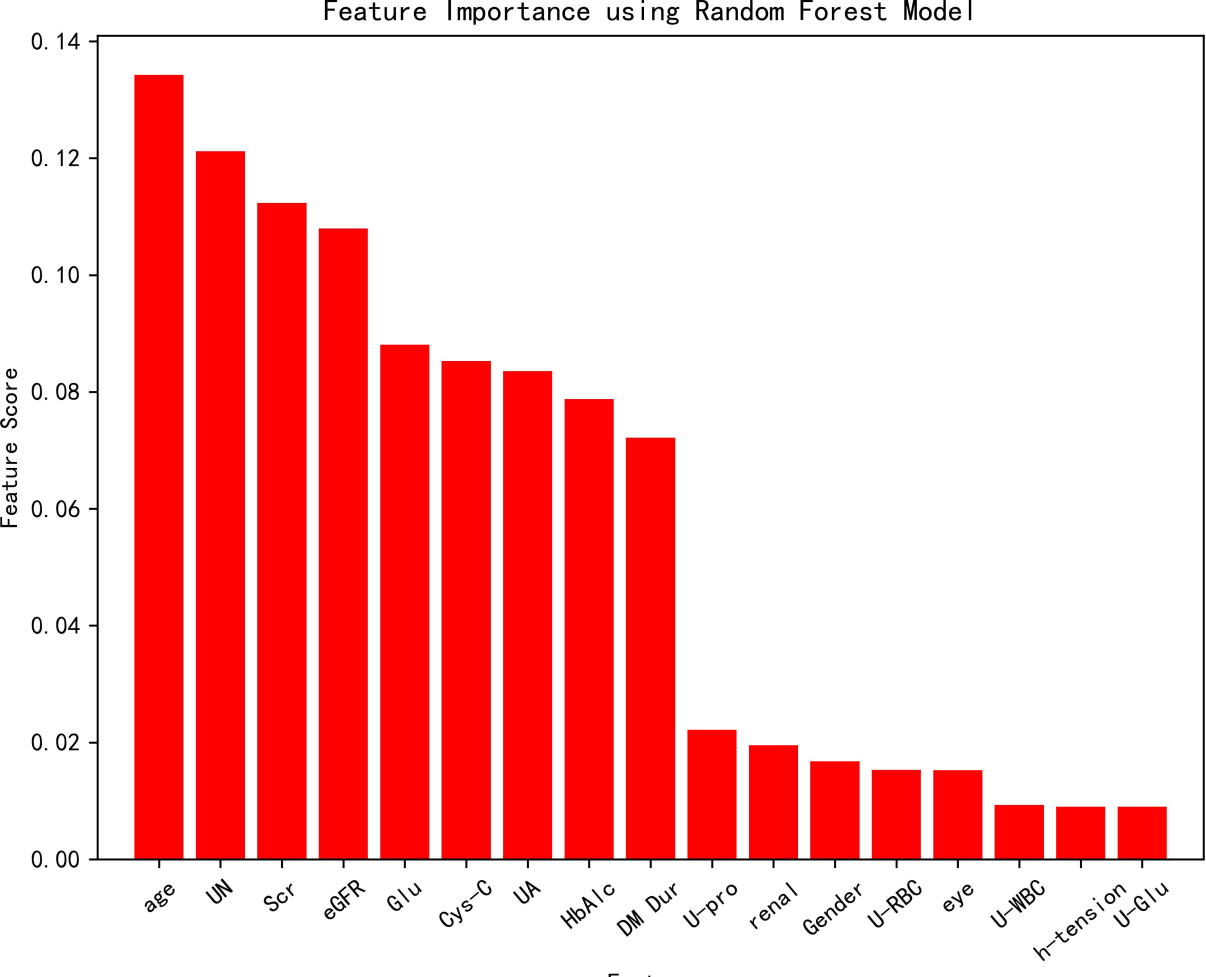
Random forest model Feature importance diagram.

**Figure 2:**
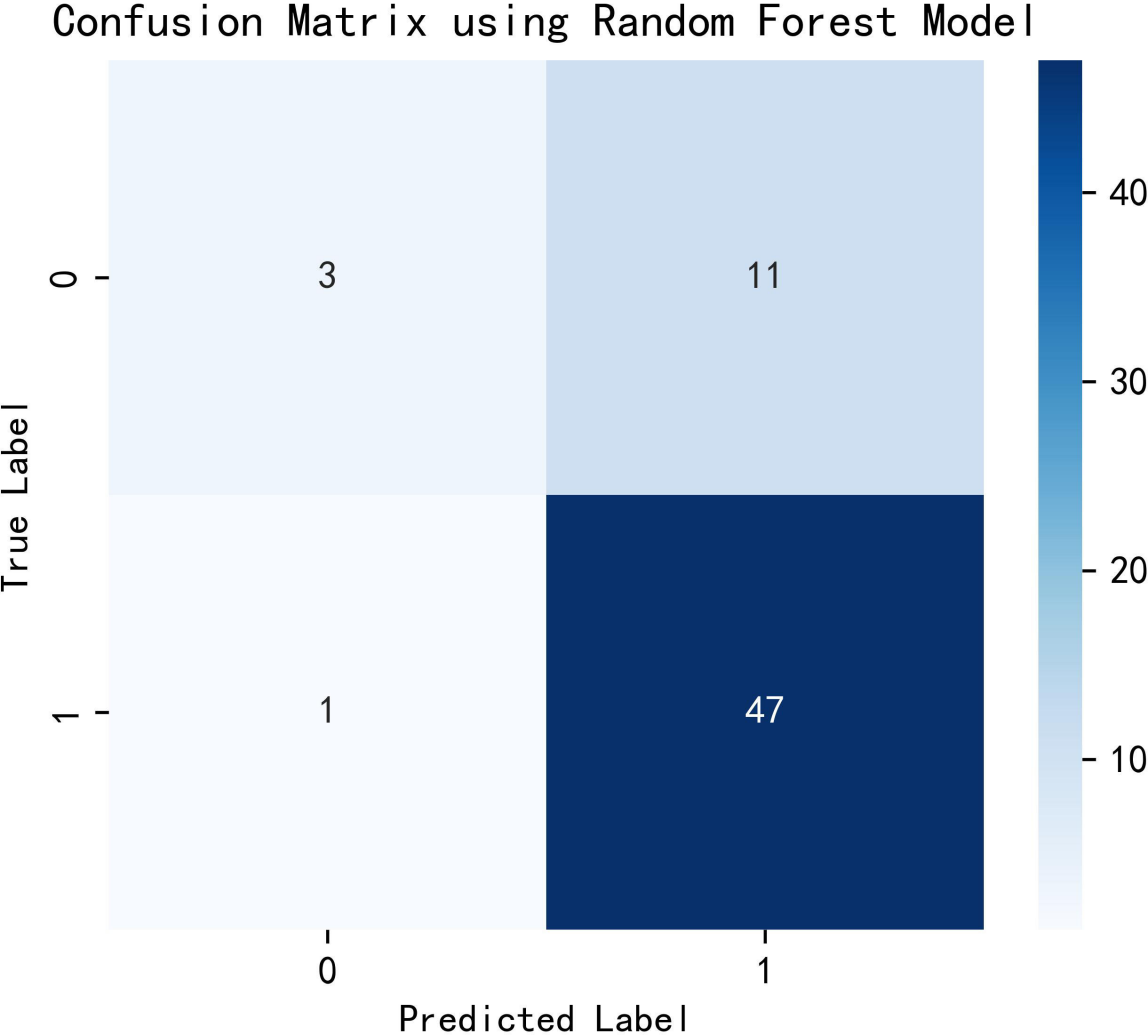
Random forest model confusion matrix heat map.

All variables were included in the random forest model, and the parameters were adjusted. The importance of each feature is ranked to obtain **Figure 1**. The constructed random forest model showed that the top five influencing factors for postoperative visual acuity improvement in working-age PDR patients undergoing vitrectomy were age, serum urea nitrogen, serum creatinine, eGFR and serum glucose. The accuracy of the prediction model is 0.81. The model appears to be superior in terms of positive prediction (**Figure 2**).

## 4 Discussion

DR is a common fundus lesion of DM which can reduce eye vision and even worsely blind. There is a certain commonality between diabetic retinopathy and many pathological processes of the microvascular system of diabetic nephropathy. Diabetic nephropathy (DN) is the main cause of chronic kidney disease and is significantly associated with the development of diabetic retinopathy. With the improvement of modern living standards and the transformation of working mode, the number of young PDR patients has increased, PDR greatly affects the vision health of the working population, there are currently few studies on young PDR patients, and there is still a great clinical appeal for evaluating the influencing factors of postoperative vision recovery, and clinical treatment measures and effect monitoring need to attract sufficient attention. This study included 306 young PDR patients treated with vitrectomy to explore the effect of baseline renal function on postoperative visual acuity in young PDR patients.

The SN-DREAMS (11) study suggested that DN is associated with visual impairment in DM patients. Patients with chronic kidney injury were more likely to develop visual impairment than those without kidney disease (nonproliferative diabetic retinopathy OR=5.01, proliferative diabetic retinopathy OR=9.7). William S Gange (12) analyzed data from 71817 newly diagnosed T2DM patients aged ≥ 18 years and showed that the incidence of PDR was higher after 5 years in patients with renal disease [OR (95% CI) = 2.68 (2.09~3.42)]. PDR progression in patients with type 2 diabetes is associated with baseline renal dysfunction, including elevated serum creatinine, decreased eGFR, and high urine albumin/creatinine ratio (13). The risk of DR was positively correlated with serum creatinine concentration, [OR (95% CI) = 1.01(1.002~1.022)] (14). Higher serum UA levels were significantly associated with adverse outcomes in DM (15). In this study, young PDR patients with reduced renal function were more likely to have visual acuity loss after vitrectomy, including high serum Cys-c levels [0.85 (0.68, 1.37) mg/L vs 0.72 (0.58, 0.88) mg/L, P=0.049], and eGFR reduction [105.70(60.67, 122.17)ml/(min/1.73m2) vs 118.99(101.97, 126.67)ml/(min/1.73m2), P=0.04]. In univariate regression analysis, the decrease in baseline eGFR and the increase in serum urea nitrogen, creatinine, and cystatin c before surgery were the contributing factors to the decline of visual acuity after vitrectomy in young PDR patients.

Saini DC (16) noted that DR may be independently associated with the occurrence of microalbuminuria and microalbuminuria is a strong predictor of kidney injury progression in DM patients. Hsieh YT’s (17) another study of 576 patients with type 2 diabetes mellitus with microalbuminuria followed for 8 years showed that remission of microalbuminuria (urine albumin/creatinine ratios < 30 mg/g at least two times in there for 6 months) was an independent protective factor for the development of PDR and DME. In this study, individuals with baseline proteinuria and hematuria had a higher rate of visual loss in young PDR patients (25.0% vs 12.9%, P=0.04; 27.1% vs 13.6%, P=0.033). Excluding other confounding factors, the risk of postoperative visual acuity loss in patients with preoperative proteinuria was higher [OR (95% CI) = 5.886 (1.101, 31.474)].

In addition, there is currently no consensus on the impact of renal impairment on the prognosis of surgical vision in PDR patients. Studies have shown that PDR patients with renal impairment are more likely to have complications and a worse prognosis after vitrectomy (18, 19). It has also been suggested that decreased renal function is not significantly associated with worsening PDR and postoperative visual impairment (20, 21). In this study, more attention was paid to young PDR patients, and 180 cases were enrolled, and the relationship between renal function and postoperative vision of PDR patients was innovatively examined from multiple dimensions such as renal history, baseline renal function serological indicators, cystatin C, eGFR and urine protein. At the same time, some limitations of the study were exposed, and follow-up studies of patients will be presented in a subsequent article.

In conclusion, understanding the factors of vision after vitrectomy in young working age PDR patients will help to the clinical therapy choice, preoperative preparation, and postoperative care of patients. Preoperative renal function and urine protein monitoring for PDR patients with risk factors, and active targeted interventions, can improve the visual acuity and quality of life of patients after vitrectomy. It is helpful to find out whether it is possible to better predict the improvement of visual acuity after vitrectomy in working-age people without adding additional preoperative testing. While saving talent and financial resources, it also ensures patient efficacy evaluation and clinical intervention.

## Data availability statement

The original contributions presented in the study are included in the article/**Supplementary Material**. Further inquiries can be directed to the corresponding author.

## Ethics statement

The studies involving humans were approved by the Ethic Committee of Hebei Eye Hospital (NO.2022KY29). The studies were conducted in accordance with the local legislation and institutional requirements. The human samples used in this study were acquired from primarily isolated as part of our previous study for which ethical approval was obtained. Written informed consent for participation was not required from the participants or the participants’ legal guardians/next of kin in accordance with the national legislation and institutional requirements.

## Author contributions

XZ: Conceptualization, Formal analysis, Investigation, Methodology, Project administration, Visualization, Writing – original draft. LF: Conceptualization, Formal analysis, Investigation, Methodology, Project administration, Visualization, Writing – original draft. CX: Data curation, Formal analysis, Software, Writing – original draft. JW: Data curation, Formal analysis, Software, Writing – original draft. WZ: Formal analysis, Supervision, Validation, Writing – original draft. FZ: Funding acquisition, Project administration, Resources, Supervision, Writing – review & editing.
